# Niche and Range Shifts of the Fall Webworm (*Hyphantria cunea* Dury) in Europe Imply Its Huge Invasion Potential in the Future

**DOI:** 10.3390/insects14040316

**Published:** 2023-03-26

**Authors:** Peixiao Nie, Rujing Yang, Runyao Cao, Xiaokang Hu, Jianmeng Feng

**Affiliations:** 1 Division of Plant Ecology, College of Agriculture and Biological Science, Dali University, Dali 671003, China; 2 Research Center for Agroecology in Erhai Lake Watershed, Division of Plant Ecology, Dali University, Dali 671003, China; 3 Cangshan Forest Ecosystem Observation and Research Station of Yunnan Province, Division of Plant Ecology, Dali University, Dali 671003, China

**Keywords:** Europe, invasion risk, niche shifts, range shifts, the fall webworm

## Abstract

**Simple Summary:**

The fall webworm (*Hyphantria cunea* Dury) has a strong impact on agricultural systems in Europe. However, its invasive potential inherited from its native niche in North America remains unknown. In the present study, we analyzed niche and range shifts of the fall webworm in Europe and compared them with those in native North America. Compared with those in Europe, the fall webworm in North America showed wider niche and larger potential ranges in Europe. Based on the invasive potential of fall webworm in Europe inherited from its native counterpart, its potential range in Europe could be 5.5-fold that of the current one. The fall webworm may prefer vast regions of Europe, excluding Norway, Sweden, Finland, North Russia, Hungary, Croatia, Romania, and Ukraine, which might be the priority regions for future invasions of the fall webworm in Europe.

**Abstract:**

The fall webworm (*Hyphantria cunea* Dury) has a strong impact on agricultural systems in Europe. However, its invasive potential, which was inherited from its native niche in North America, remains unknown. Here, we investigated the climatic niche and range shifts of the fall webworm in Europe and compared them with those in native North America, then assessed the worms’ invasive potential in Europe. Compared with the fall webworm in Europe, those in North America survived in more diverse climatic conditions, which was closely associated with their broader niche and larger potential ranges in Europe. If the fall webworm in Europe could exploit the native niche inherited from those in North America to adapt to climatic conditions in Europe, their potential ranges in Europe could be 5.5-fold those based on the niche as introduced in Europe. The potentially unfilled ranges of the fall webworm in Europe were mainly detected in vast regions of Europe, excluding Norway, Sweden, Finland, North Russia, Hungary, Croatia, Romania, and Ukraine, suggesting that, without strict control, these vast regions might be preferably invaded by the fall webworm in Europe in the future. Therefore, strict control against its invasion is needed. Given that small niche shifts in this invasive insect could result in large range shifts, the niche shifts represent a more sensitive indicator of invasion risk than range shifts.

## 1. Introduction

The spread of alien invasive species is one of the major drivers of global change, triggering impacts on global biodiversity, ecosystem and human welfare [1,2,3]. Among all types of invaders, alien invasive insects have received much attention, probably due to their close association with the dissemination of human disease [4,5,6], spread of plant pathogens and huge impact on global agriculture [7]. Projecting the invasive potentials and ranges of alien invasive insects may facilitate the identification of priority regions and development of strategies against their invasions [8,9,10].

The adaptability of alien invasive species to environmental conditions is linked to their invasive potential, with stronger adaptability to diverse environmental conditions linked to greater potential invasion [11,12]. Notably, the niche conception, which describes the environmental survival and adaptation of a specific species [13], has been widely used because niche breadth partly reflects the invasive potential [14]. Accordingly, ecological niche models (ENMs) have been frequently used to project potential ranges of alien invaders [15,16,17]. In these ecological niche models, scientists approximated the realized niche of the alien invasive species by correlating their occurrences with environmental predictors [18]. In our study, we assumed that, due to relatively short introduction history and control measures, the niche breadth of alien invasive species in the introduced regions may be narrower than that of its native counterparts. Therefore, if it could exploit its native niche to adapt to the climatic conditions in the introduced regions, its invasive potential might be enhanced.

To detect priority regions for controlling biological invasions under current and future scenarios, the projection of the potential ranges of alien invasive species has received substantial attention [8,9,10]. For example, under future climate scenarios, some countries in America and East Asia might be preferred for the implementation of strategies against the invasion of a tropical fire ant (*Solenopsis geminate*, Hymenoptera: Formicidae) [19]. Interestingly, range shifts of alien invasive species are closely related to their niche shifts, and numerous studies have reported range expansions of the invaders with increased niche breadth [20,21]. In the present study, we hypothesized that alien invasive species need a long time period to fully exploit their native niches, and therefore their current potential ranges may not be larger than those of their native counterparts in the introduced regions.

As a highly polyphagous pest from North America, the fall webworm (FW, *Hyphantria cunea* Dury) belongs to the order Lepidoptera, family Erebidae. Its high polyphagy is characterized by its feeding on more than 600 plant species, such as forest trees, fruit trees, herbs, agricultural crops and shrubs, with a preference for hickories, walnuts, alders, mulberries, sweetgums and poplars [22]. In addition, its high ecological flexibility and strong adaptability to diverse environmental conditions seriously hinder its control [23]. Since its introduction of Europe in 1940s, the continent has become a major invasion hotspots. This moth has invaded nearly twenty European countries, causing huge economic damage, primarily to fruit trees, ornamental plants and forest trees [24,25,26]. It has become a notorious agricultural pest in Europe [26,27,28]. Therefore, it is critical to understand its invasive potentials and potential ranges in Europe.

Climatic conditions, especially temperature, are one of the key factors in insect biology and ecology [29]. Accordingly, one of the most important determinants of this insect’s successful survival is its strong adaptability to low temperature climatic conditions [29,30]. In North America, the distribution of the fall webworm ranges from approximately 19° to 55° north latitude in North America, whereas in Europe, its distribution ranges from 37° to 55° north latitude [26,31,32,33,34]. Thus, relative to its native counterpart in North America, the fall webworm in Europe may not show wider climatic niche breadth, especially in temperature. Recently, Tang et al. (2021) detected low overlap between climatic niches of the fall webworm in North America and China [35]. However, to our knowledge, the niche shifts between the fall webworm in Europe and North America have yet to be reported.

As discussed above, projecting the potential ranges of alien invasive species is one of the hotspots in invasion biology. Thus, similar to other alien invasive species, the potential ranges of fall webworm have attracted substantial attention. For example, Ge et al. (2018) projected that, under the current climate scenario, this insect could survive on all of five continents. Temperature-related factors were primarily responsible for its spatial distribution patterns, whereas under future climate change scenarios, its potential ranges could increase in middle and high latitude regions and decrease in the low areas [36]. Additionally, Tang et al. (2021) found range expansions of this invasive insects in both China and the United States of America under future climate change scenarios [35]. Undoubtedly, these studies have enriched our knowledge about controlling the invasion of this species. However, these studies generally investigated the insect’s range shifts under the scenarios of future climate changes. There is an un-testified hypothesis that the fall webworm in Europe has not fully occupied its native niche or adapted compared with its native counterpart, and therefore its climatic potential range in Europe might not be larger than that of fall webworm in North America.

In our study, the niche and range dynamic models were applied to examine the climatic niche and range shifts between the fall webworm in Europe and those in North America and the underlying mechanisms. Additionally, we examined the potentially unfilling ranges of the fall webworm in Europe which could be potential invasion sites in the future. The present study, from a climate suitability perspective, sheds light on our understanding of insect invasion into Europe for possible control.

## 2. Materials and Methods

### 2.1. Occurrence of Fall Webworm

**Figure 1:**
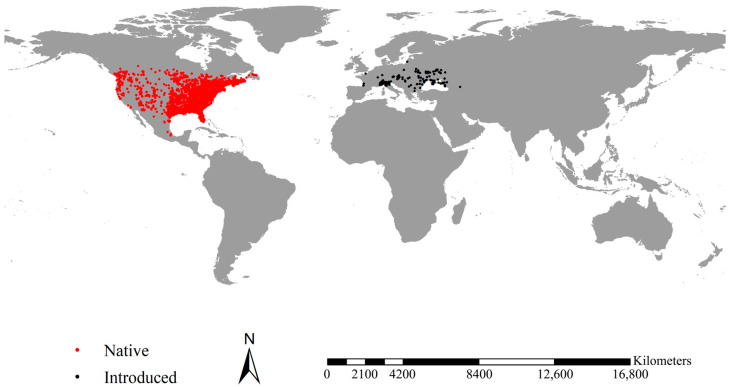
Occurrence records of the fall webworm. Black and red points represent the occurrence of the fall webworm in North America and the fall webworm in Europe, respectively. After spatial sparsification, we obtained a total of 3661 records, including 3465 records of the fall webworm in North America and 196 records of the fall webworm in Europe.

We retrieved occurrence records of the fall webworm from the Global Biodiversity Information Facility (GBIF), the world largest network and data infrastructure carrying records of more than two billion species, including more than 80,000 datasets and more than 8000 peer-reviewed papers. We initially obtained 16,398 occurrence records of the fall webworm. Then, we deleted those with geographical coordinate uncertainty greater than 10 km. To reduce the bias associated with spatial autocorrelation, we spatially reduced the occurrence with a 5 km radius using a species distribution model [37,38]. Finally, we built a dataset comprising 196 and 3465 records of the fall webworm in Europe and those in North America, respectively (Figure 1, Online dataset 1).

### 2.2. Climatic Predictors

Climatic predictors used in the present study were retrieved from Worldclim 2.1, a widely accepted global climate dataset [39]. As most studies suggested [35,36], we adopted datasets of near-current conditions (1970–2000) to explore climatic niche and range dynamics of the fall webworm in Europe and in North America. The datasets comprise 19 climatic predictors, including 11 temperature-related predictors and 8 precipitation-related ones. Additionally, the 19 climatic predictors not only include annual and monthly predictors (e.g., mean annual temperature and precipitation in the wettest month), but also seasonal ones (e.g., temperature seasonality and mean temperature in winter). The spatial resolution of the climatic predictors in the present study was 5 arc-minute (ca. 10 km).

### 2.3. Niche Shifts between the Fall Webworm in Europe and North America

Based on the Ecospat *R* package developed by Di Cola et al. (2017) [40], we applied the Centroid shift, Overlap, Unfilling and Expansion (COUE) scheme, a niche dynamic model developed by Broennimann et al. (2012) [41] and improved by Petitpierre et al. (2012) [42], to investigate niche shifts between the fall webworm in Europe and in North America. First, we extracted values of 19 climatic predictors for each occurrence record of the fall webworm in Europe and in North America. Second, we conducted principal component analysis (PCA) to generate first and second principal component axes, and also identified the major predictors responsible for first and second principal component axes. The total climatic niche space delimited by the first and second principal component axes was transformed into a grid layer with 100 × 100 cells, and the kernel density function was used to determine the smoothed density of occurrences [18]. Third, the overall niche space was decomposed into three elements: niche stability (NS), niche unfilling (NU), and niche expansion (NE) [18,41]. Niche stability indicates the niche space shared by the fall webworm in Europe and North America. Niche unfilling represents the niche space exploited only by the fall webworm in North America. Niche expansion is the niche space used only by the fall webworm in Europe. Niche breadth of the fall webworm in North America is the sum of niche unfilling and niche stability; niche breadth of the fall webworm in Europe is the sum of niche expansion and niche stability. Niche breadth ratio (*BR*) [43] represents the ratio of the climatic niche breadth of the fall webworm in North America (*NBN*) to that of the fall webworm in Europe (*NBI*), which was calculated as follows:

$$\mathrm{BR}\;=\;\frac{\mathrm{NBN}}{\mathrm{NBI}}$$

The ratio of the climatic niche breadth > 1 indicated that the climatic niche breadth of the fall webworm in Europe was narrower than that of those in North America and vice versa.

We estimated the niche similarity index (*NSI*) [44] to determine the shifts of niche position between the fall webworm in Europe and North America, as follows:

$$\mathrm{NSI}\;=\;\frac{2\mathrm{NS}}{\mathrm{NBN}\;+\;\mathrm{NBI}}$$

If niche similarity index < 0.5, the fall webworm in Europe and North America occupied different niche positions.

Additionally, as suggested by Yang et al. (2023) [45], paired samples’ *t* tests were conducted to compare the ranges of 19 predictors of occurrences associated with the fall webworm in Europe and those in North America.

### 2.4. Projecting Potential Ranges of the Fall Webworm in Europe and Those in North America

We used the occurrence records of the fall webworm in Europe and those in North America and the 19 spatial layers of climatic predictors from Worldclim 2.1 [39] to project potential ranges of the fall webworm in Europe and North America, and then in Europe. Notably, for the potential ranges of the North American fall webworm in Europe, we developed ecological niche models for the North American fall webworm in North America, then projected its potential range in Europe.

To reduce the effects of collinearity among the predictors on ecological niche models, we built preliminary ecological niche models to separately calibrate the importance of predictors for the fall webworm in Europe and those in North America (Supplementary Material Table S1). We also conducted Pearson correlation analysis to detect collinearity among the predictors (Supplementary Material Table S2). As suggested by Dormann et al. (2013) [46], the threshold of collinearity was Pearson correlation coefficient ≥ 0.7. In each pair of climatic predictors that showed collinearity, we retained the predictors with higher importance values (Supplementary Material Table S3). We repeated this process until no collinearity was detected among the predictors. We adopted biomod2, an assembled ecological niche model platform developed by Thuiller et al. (2009) [15], to project potential ranges of the fall webworm in Europe and those in North America. As most studies suggested, we used seven algorithms in our ecological niche models, including the artificial neural network, generalized additive model, flexible discriminant analysis, random forest classifier, classification tree analysis, generalized boosting model, and maximum entropy model [47,48]. Based on the study of Barbet-Massin et al. (2012) [49], we generated pseudo-existence records (PAs) for each ecological niche model. We randomly generated 1000 pseudo-existence records for the fall webworm in Europe’s ecological niche models because there were less than 1000 records, and 3465 pseudo-existence records (equal to the number of the fall webworm in North America records) were randomly generated because the number of the fall webworm in North America records was greater than 1000.

To evaluate the reliability of ecological niche models, a five-time cross-validation was utilized. In this cross-validation, 30% of the records were used to assess the models’ performance, and the remaining 70% were used to develop ecological niche models [48]. Following Gallien et al. (2012) [50], we only retained ecological niche models with true skill statistics (TSS) [51] > 0.7, or an area under the receiver operating characteristic curve (AUC) [52] > 0.8. We utilized the sensitivity–specificity sum maximization approach (MSS threshold) [53] to calibrate potential ranges of the fall webworm in Europe and in North America.

### 2.5. Range Shifts between the Fall Webworm in Europe and Those in North America

We used a range dynamic model developed by Yang et al. (2023) [45] to analyze the range shifts between the fall webworm in Europe and those in North America. In this model, the total potential range of the fall webworm in Europe and those in North America was divided into three components: expanded range (*ER*), stabilized range (*SR*) and unfilling range (*UR*) [45]. In our study, expanded range was the range only potentially exploited by the fall webworm in Europe; stabilized range indicated the range potentially exploited by the fall webworm in Europe and those in North America; unfilling range was the range only potentially exploited by the fall webworm in North America. Accordingly, the potential range of the fall webworm in Europe is the sum of expanded range and stabilized range, whereas that of the fall webworm in North America is the sum of unfilling range and stabilized range. We built an index of range ratio (*RR*) [45] used to compare the ranges between the fall webworm in Europe (*RI*) and those in North America (*RN*), as follows:

$$\mathrm{RR}\;=\;\frac{\mathrm{RN}}{\mathrm{RI}}$$

when range ratio < 1, the range of the fall webworm in Europe was larger than that of those in North America and vice versa.

Additionally, we built an index of range similarity (*RSI*) [45] to measure the shifts in range between the ranges of the fall webworm in Europe and in North America, as follows:

$$\mathrm{RSI}\;=\;\frac{\mathrm{SR}}{\mathrm{RI}\;+\;\mathrm{RN}}$$

when range similarity < 0.5, the fall webworm in Europe and those in North America were in different ranges and vice versa.

## 3. Results

### 3.1. Climatic Predictors Responsible for the Niche Dynamics

**Figure 2:**
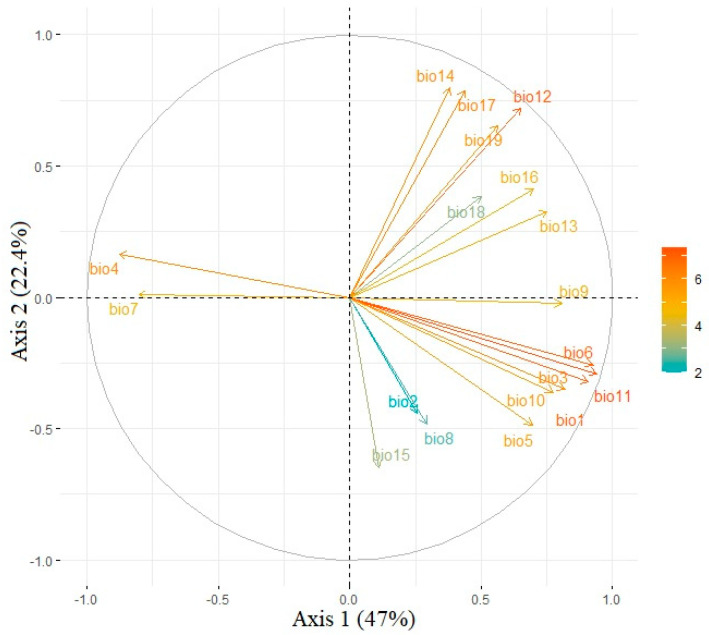
Principal component analysis used to generate axes for delimiting niche spaces. Load of each predictor is indicated by graduated colors. The first and second principal component axes explain 69.4% of the variations among the predictors, and the first and second principal component axes were responsible for 47.0% and 22.4% of the variations, respectively. bio1: annual mean temperature; bio2: mean diurnal range; bio3: isothermality; bio4: temperature seasonality; bio5: maximum temperature of warmest month; bio6: minimum temperature of coldest month; bio7: temperature annual range; bio8: mean temperature of wettest quarter; bio9: mean temperature of driest quarter; bio10: mean temperature of warmest quarter; bio11: mean temperature of coldest quarter; bio12: annual precipitation; bio13: precipitation of wettest month; bio14: precipitation of driest month; bio15: precipitation seasonality; bio16: precipitation of wettest quarter; bio17: precipitation of driest quarter; bio18: precipitation of warmest quarter; bio19: precipitation of coldest quarter.

**Figure 3:**
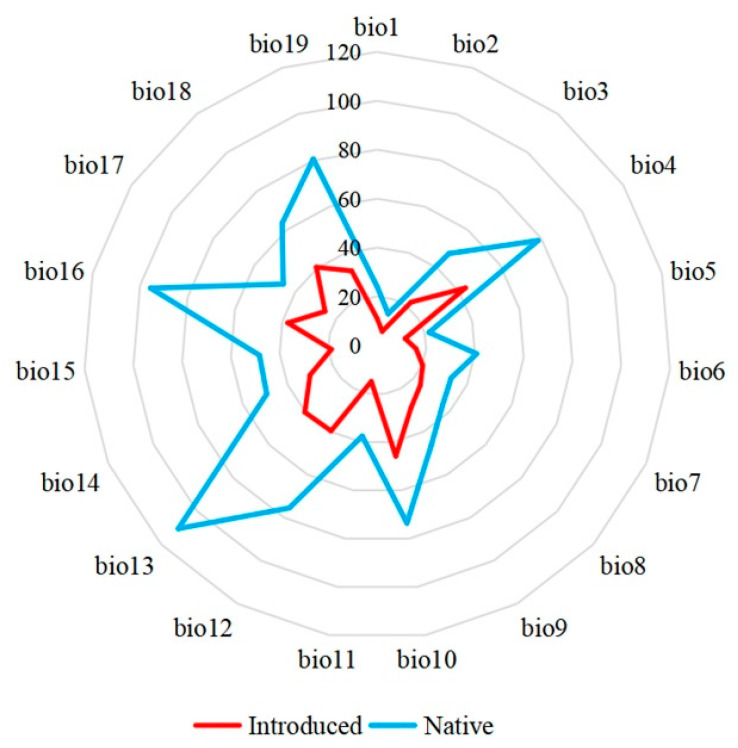
Comparisons of the ranges of the 19 predictors between those extracted from the occurrences of the fall webworm in Europe and the fall webworm in North America. Paired-samples *t*-tests showed that the ranges of the fall webworm in North America were significantly larger than those of the fall webworm in Europe (*p* = 0.01). The abbreviations are as indicated in the Figure 2 legend.

Our principal component analysis showed that the first and second principal component axes totally explained 69.4% of the variations among the 19 climatic predictors. The first principal component axis was responsible for 47.0% of the variations with the mean temperature in the winter season showing the highest load (0.94), followed by the minimum temperature of the coldest month (0.93) and the annual mean temperature (0.91) (Figure 2). Therefore, the first principal component axis was mainly associated with environmental energy. The second principal component axis explained 22.4% of the variations, with the precipitation in the driest month associated with the highest load (0.80), followed by precipitation of the driest quarter (0.79) and annual precipitation (0.72) (Figure 2). Therefore, the second principal component axis was primarily associated with the water available in the environment. In sum, compared with the water-related predictors, temperature-related ones might play a more important role in the niche shifts between the fall webworm in Europe and those in North America. Additionally, the paired samples *t* test showed that ranges of all 19 climatic predictors extracted from the occurrence of the fall webworm in North America were significantly wider than those extracted from the occurrence of the fall webworm in Europe (*P* = 0.01, Figure 3). Notably, 69.4% of the variation in the principal component analysis may suggest that nearly 30% of the variation was unexplained, which may be due to the two dimensions of the climatic niche space considered in this study.

### 3.2. Niche Dynamics of the Fall Webworm in Europe and Those in North America

**Figure 4:**
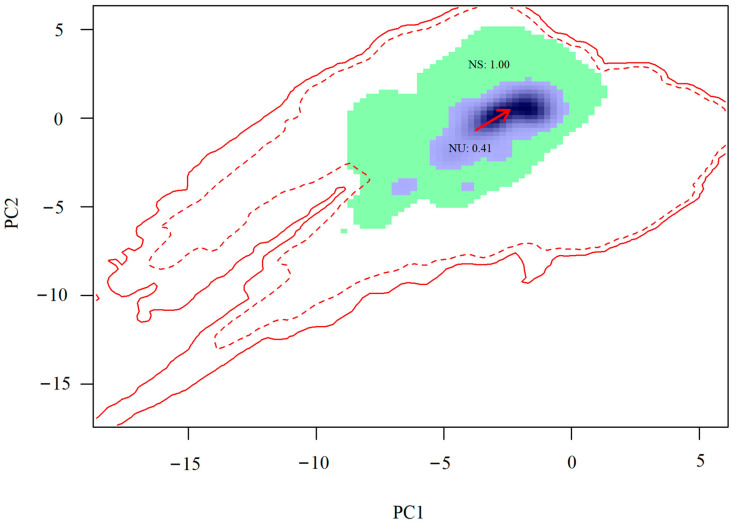
Niche shifts between the fall webworm in Europe and the fall webworm in North America. The green and blue colors indicate niche stability and unfilling niche, respectively, which were 1.00 and 0.41, respectively. Red arrow represents the direction of niche shifts. Solid and dotted contour lines in red indicate climatic conditions of the fall webworm in North America and Europe, respectively.

According to the Centroid Shift, Overlap, Unfilling and Expansion scheme, the niche expansion of the fall webworm in Europe relative to that in North America was 0.00 (Figure 4). The stabilized niche between them was 1.00, and that of unfilling niche was 0.41 (Figure 4). Accordingly, the niche breadths of the fall webworm in Europe and that in North America were 1.00 and 1.41, respectively. The niche breadth ratio was 0.701, suggesting that the niche breadth of the fall webworm in North America was approximately 1.41 times that of the fall webworm in Europe. Thus, the fall webworm in Europe has not potentially exploited 29.1% of the niches of the fall webworm in North America. The niche similarity index was 0.83, indicating that the two fall webworm occupy similar niches. In sum, the niche of the fall webworm in Europe was stabler than that of the fall webworm in North America, without further niche expansion beyond the native niche and small shifts in niche positions.

### 3.3. Potential Ranges of the Fall Webworm in Europe and Those in North America

**Figure 5:**
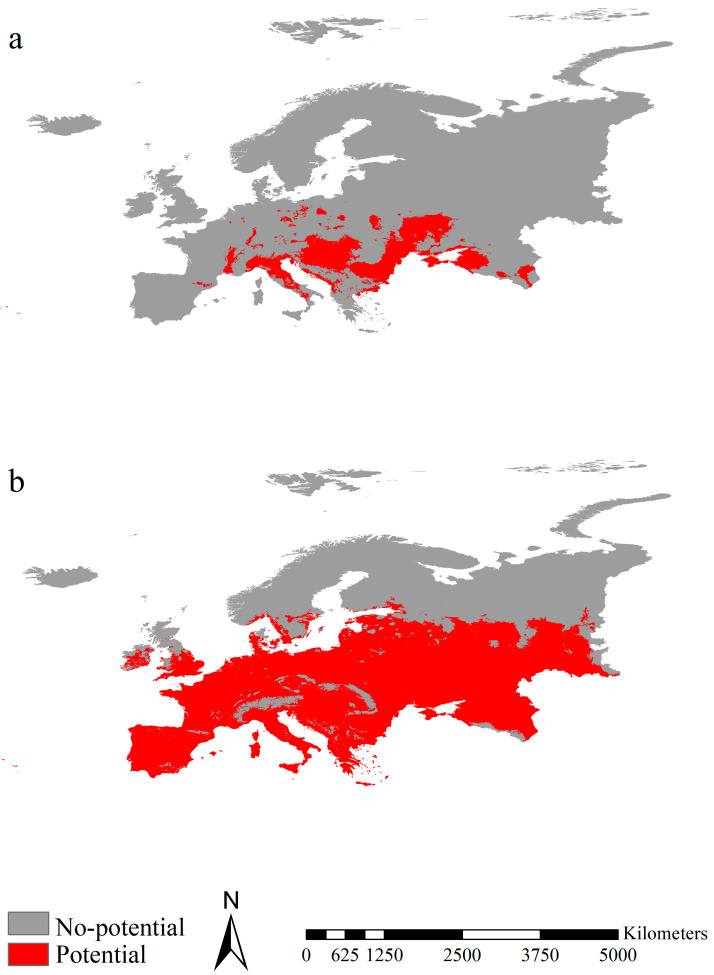
Potential ranges of the fall webworm. (**a**,**b**) indicate the potential ranges of the fall webworm in Europe and the fall webworm in North America, respectively. The potential ranges for the fall webworm in Europe were mainly projected in France, Austria, Hungary, Croatia, Romania, Bulgaria and Ukraine. The potential ranges for the fall webworm in North America were mostly identified in vast regions of Europe, excluding Norway, Sweden, Finland and North Russia.

The assembled ecological niche models generated by the seven algorithms for the fall webworm in Europe and in North America showed high reliability with the true skill statistics (area under the curve) values of 0.834 (0.972) and 0.815 (0.964), respectively. The sensitivity–specificity sum maximization approach thresholds used to determine the potential ranges in Europe for the two fall webworm were 0.51 and 0.31, respectively. The potential ranges of the fall webworm in Europe were mainly detected in France, Austria, Hungary, Croatia, Romania, Bulgaria and Ukraine, covering 1,006,508 km^2^ (Figure 5a). Potential ranges of the fall webworm in North America were detected in vast regions of Europe, excluding Norway, Sweden, Finland and North Russia, covering 5,536,792 km^2^, i.e., more than 50% of the area of Europe (Figure 5b).

### 3.4. Range Dynamics between the Fall Webworm in Europe and North America

**Figure 6:**
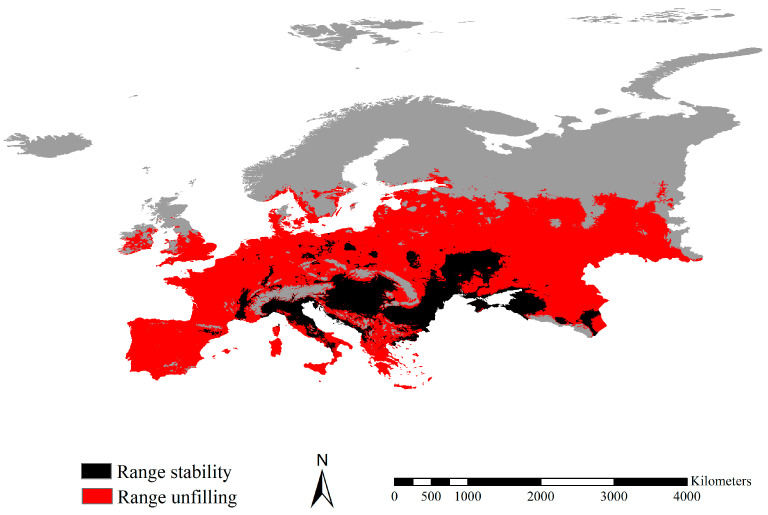
Range dynamics of the fall webworm in Europe and in North America. The red and black indicate unfilling and stabilized ranges, respectively. Range ratio and range similarity were ca. 5.50 and 0.31, respectively. The climatic potential ranges for the fall webworm in North America, but not the fall webworm in Europe, were mostly projected in vast regions of Europe, excluding Norway, Sweden, Finland, North Russia, Hungary, Croatia, Romania, and Ukraine. The climatic potential range for the fall webworm in both Europe and in North America was mostly identified in France, Austria, Hungary, Croatia, Romania, Bulgaria and Ukraine.

Our range dynamic analysis showed that the range expansion of the fall webworm in Europe relative to the fall webworm was 0.00. The stabilized range shared by the two webworm was 1.00, mainly projected in Hungary, Croatia, Romania, and Ukraine, covering 1,006,508 km^2^ (Figure 6). The potentially unfilling range of the fall webworm in Europe relative to the fall webworm in North America was 4.50, mainly detected in vast regions of Europe, excluding Norway, Sweden, Finland, North Russia, Hungary, Croatia, Romania, and Ukraine, covering 4,530,283 km^2^ (Figure 6). The ratio of potential ranges of the fall webworm in North America to those of the fall webworm in Europe was ca. 5.50. Thus, the fall webworm in Europe has yet to potentially exploit 84.5% of the potential ranges of the fall webworm in North America. The range similarity index of 0.31 suggests that they occupy different range positions and large shifts in niche positions. In sum, the potential ranges of the fall webworm in Europe were relatively stable, without range expansions beyond the potential ranges of the native fall webworm in the North America population.

## 4. Discussion

Our observations indicated that the fall webworm in North America had wider niche breadth (approximately 1.41-fold) and larger potential range (nearly 5.5-fold) in Europe than the fall webworm in Europe. The fall webworm in Europe has not exploited or occupied 29.1% of the niches and 84.5% potential ranges of fall webworm in North America, its native counterpart. It may imply that, if the fall webworm in Europe utilized its inherited niche to adapt to climatic conditions in Europe, its climatic potential ranges in future could be 5.5-fold its current potential ranges, suggesting the need for strict control against insect invasion.

Range shifts have been frequently used to evaluate the risk of invasion by alien species [35,36,54]. In most cases, this risk indicator is generally positively and closely associated with niche shifts [21,55]. In our study, the fall webworm in North America had larger climatic potential range (nearly 5.5-fold) in Europe relative to the fall webworm in Europe, whereas the niche breadth of the fall webworm in North America was nearly 1.41-fold the niche breadth of the fall webworm in Europe. Additionally, small shifts in niche position could result in substantial shift in the range of the fall webforms in Europe and in North America (niche similarity index: 0.83 vs. range similarity index: 0.31). These observations may imply that small niche shifts trigger large range shifts, which is supported by recent findings [45,48,56]. Therefore, although from a climate suitability perspective, both the climatic niche shifts and potential range shifts of invasive species can be used to assess the invaders’ risk, niche shifts are a more sensitive indicator than range shifts, and invasive species showing niche shifts require further attention.

Our results showed that the ranges of all 19 climatic predictors for the fall webworm in North America were considerably and significantly larger than those for the fall webworm in Europe, which was consistent with the greater distribution of the fall webworm in North America across latitude [32,33,34]. Our observations indicated that the occurrence of the fall webworm in Europe was only recorded in Italy, Hungary, Bulgaria, and Romania, whereas the fall webworm in North America was detected in across almost all of the United States of America. This range may suggest that the fall webworm in Europe does not strongly adapt to the diverse climatic conditions which are conducive to fall webworm in North America. It also might be closely associated with narrower niche breadth and smaller potential ranges of the fall webworm in Europe relative to the North America fall webworm in Europe. Additionally, our principal component analysis showed that mean temperature in the winter season and the precipitation in the driest month played the strongest roles in the niche dynamics. Thus, compared with other climatic factors, the smaller ranges of these two climatic factors for the fall webworm in Europe have a stronger influence on its narrower niche breadth and smaller potential ranges.

In our study, the fall webworm in Europe showed a narrower niche breadth and smaller potential ranges relative to its native counterpart (fall webworm in North America), which might be linked to various factors. First, the fall webworm was introduced into Europe only 80 years ago [57,58], whereas fall webworm in North America evolved over millions of years to develop and enlarge its niche and range, as well as to acclimatize to diverse climatic conditions [59,60]. Therefore, compared with the fall webworm in North America, those in Europe had less time to develop and expand niches and potential ranges. Second, the fall webworm in North America was not an alien insect in North America, and measures to control its spread and proliferation were taken long after its evolution. The fall webworm in Europe, however, was rated as a quarantine alien invasive insect shortly after its introduction into Europe [57,58]. In summary, the time between its introduction into Europe and the control measures might be associated with the narrower niche breadth and smaller potential ranges of the fall webworm in Europe compared with the fall webworm in North America, though other factors, such as host plant monocultures [61] and predation pressure [62], may also play an important role.

Ge et al. (2018) observed that future climate changes could facilitate the survival of this invasive insect in most parts of the world, and that the expansion of its potential ranges could be detected in middle and high latitudes [36]. Additionally, a recent study by Tang et al. (2021) showed that this invasive insect in China and the United States had small niche overlap, and range expansions in future could occur in both China and the United States [35]. These studies further our understanding of the invasive mechanisms of this insect at global and national levels. By contrast, the present study applied niche and range dynamic models to investigate the niche and range shifts between the two webworm. We not only calibrated the unfilling niche, which the fall webworm in Europe could exploit in future, but also detected potential ranges of invasion in Europe by the fall webworm.

Our study showed that the potential ranges of the fall webworm in Europe were roughly centered around Hungary, the region through which this invasive moth was initially introduced into Europe [57,58]. As discussed above, the proliferation or invasion of this moth in Europe might be constrained by the relatively short time between its introduction and quarantine. This is consistent with the geographical center of the occurrence of the fall webworm in Europe that is reported in the present study. Thus, the prevailing climatic conditions in the regions around Hungary primarily shape the niches of the fall webworm in Europe and its potential ranges in Europe. Therefore, the niches and potential ranges of the fall webworm in Europe were primarily determined by the prevailing climatic conditions in the regions where it was initially introduced, and the relatively short time between its introduction and control.

Our observations showed that the potential ranges of the fall webworm in Europe were mainly detected in France, Austria, Hungary, Croatia, Romania, Bulgaria and Ukraine, covering 1,006,508 km^2^. It may suggest the need for strict control of this invasive insect in these priority regions. Our range shift analysis also showed that the potentially unfilling ranges of the fall webworm in Europe were mainly detected in the vast regions of Europe, excluding the northern regions (Norway, Sweden, Finland, North Russia, Hungary, Croatia, Romania, and Ukraine) and eastern areas (Hungary, Croatia, Romania, and Ukraine), spanning 4,530,283 km^2^. This finding may suggest the need for precautions against its invasion into these regions in future.

## 5. Conclusions

The wider native niche relative to that of Europe may suggest that the fall webworm in Europe may show stronger adaptability to climatic conditions in future than under current conditions, suggesting possible expansion of its range of climatic potential. We also detected the potentially unfilling ranges of the fall webworm in Europe relative to its native counterpart in North America. This might be the priority region for future invasions of the fall webworm in Europe. Additionally, small shifts between the two webworm may trigger a large shift in range. Compared with range shifts, niche shifts play a greater role in risk assessment of this invasive moth.

## Data Availability

The data (Online dataset 1) that support the findings of this study are available at https://doi.org/10.6084/m9.figshare.22060496.v1 accessed on 1 October 2022.

## Supplementary Materials

The following supporting information can be downloaded at: https://www.mdpi.com/article/10.3390/insects14040316/s1, Table S1: Importance values of climatic predictors in the preliminary ecological niche models; Table S2: Pearson correlations among the nineteen climatic predictors; Table S3: The predictors in the final ecological niche models.
