# Supplementary material for: Niche and Range Shifts of the Fall Webworm (Hyphantria cunea Dury) in Europe Imply Its Huge Invasion Potential in the Future
Source: Insects. 2023 Mar 26;14(4):316. doi: 10.3390/insects14040316 (PMC10141053; doi:10.3390/insects14040316)
Supplement: Supplementary file 1 [file insects-14-00316-s001.zip › Table S1.pdf]

Table S1. Importance values of climatic predictors in the preliminary ecological niche models.

Hyphantria cunea in North America		*Hyphantria cunea* in Europe	
Predictors	Importance values	Predictors	Importance values
bio1	0.19	bio1	0.26
bio2	0.06	bio2	0.09
bio3	0.08	bio3	0.14
bio4	0.07	bio4	0.21
bio5	0.14	bio5	0.16
bio6	0.14	bio6	0.18
bio7	0.06	bio7	0.25
bio8	0.01	bio8	0.04
bio9	0.02	bio9	0.04
bio10	0.35	bio10	0.53
bio11	0.32	bio11	0.27
bio12	0.09	bio12	0.11
bio13	0.03	bio13	0.08
bio14	0.04	bio14	0.06
bio15	0.07	bio15	0.05
bio16	0.08	bio16	0.11
bio17	0.12	bio17	0.10
bio18	0.02	bio18	0.09
bio19	0.05	bio19	0.09
