# Supplementary material for: Niche and Range Shifts of the Fall Webworm (Hyphantria cunea Dury) in Europe Imply Its Huge Invasion Potential in the Future
Source: Insects. 2023 Mar 26;14(4):316. doi: 10.3390/insects14040316 (PMC10141053; doi:10.3390/insects14040316)
Supplement: Supplementary file 1 [file insects-14-00316-s001.zip › Table S2.pdf]

Table S2. Correlations of the predictors in *Hyphantria cunea* in North America (N=3465).

		bio1	bio10	bio11	bio12	bio13	bio14	bio15	bio16	bio17	bio18	bio19	bio2	bio3	bio4	bio5	bio6	bio7	bio8	bio9	Note
bio1	Pearson Correlation	1	.950**	.978**	.355**	.497**	.159**	.176**	.410**	.214**	.376**	.222**	.331**	.780**	-.800**	.899**	.956**	-.731**	.504**	.723**	
	Sig. (2-tailed)		.000	.000	.000	.000	.000	.000	.000	.000	.000	.000	.000	.000	.000	.000	.000	.000	.000	.000	
bio10	Pearson Correlation	.950**	1	.868**	.251**	.333**	.132**	.092**	.242**	.186**	.351**	.060**	.349**	.611**	-.576**	.961**	.829**	-.503**	.577**	.594**	
	Sig. (2-tailed)	.000		.000	.000	.000	.000	.000	.000	.000	.000	.000	.000	.000	.000	.000	.000	.000	.000	.000	
bio11	Pearson Correlation	.978**	.868**	1	.390**	.560**	.163**	.217**	.481**	.214**	.344**	.316**	.317**	.852**	-.906**	.820**	.991**	-.839**	.419**	.776**	
	Sig. (2-tailed)	.000	.000		.000	.000	.000	.000	.000	.000	.000	.000	.000	.000	.000	.000	.000	.000	.000	.000	
bio12	Pearson Correlation	.355**	.251**	.390**	1	.794**	.773**	-.332**	.826**	.818**	.636**	.847**	-.223**	.246**	-.431**	.093**	.414**	-.530**	-.097**	.462**	
	Sig. (2-tailed)	.000	.000	.000		.000	.000	.000	.000	.000	.000	.000	.000	.000	.000	.000	.000	.000	.000	.000	
bio13	Pearson Correlation	.497**	.333**	.560**	.794**	1	.291**	.264**	.980**	.346**	.505**	.719**	-.078**	.507**	-.638**	.219**	.576**	-.673**	.030	.534**	
	Sig. (2-tailed)	.000	.000	.000	.000		.000	.000	.000	.000	.000	.000	.000	.000	.000	.000	.000	.000	.076	.000	
bio14	Pearson Correlation	.159**	.132**	.163**	.773**	.291**	1	-.769**	.328**	.983**	.521**	.602**	-.226**	-.012	-.158**	-.010	.178**	-.263**	-.177**	.287**	
	Sig. (2-tailed)	.000	.000	.000	.000	.000		.000	.000	.000	.000	.000	.000	.475	.000	.575	.000	.000	.000	.000	
bio15	Pearson Correlation	.176**	.092**	.217**	-.332**	.264**	-.769**	1	.214**	-.751**	-.192**	-.205**	.281**	.406**	-.277**	.166**	.196**	-.164**	.178**	.101**	
	Sig. (2-tailed)	.000	.000	.000	.000	.000	.000		.000	.000	.000	.000	.000	.000	.000	.000	.000	.000	.000	.000	
bio16	Pearson Correlation	.410**	.242**	.481**	.826**	.980**	.328**	.214**	1	.379**	.535**	.755**	-.134**	.445**	-.586**	.122**	.505**	-.640**	-.014	.484**	
	Sig. (2-tailed)	.000	.000	.000	.000	.000	.000	.000		.000	.000	.000	.000	.000	.000	.000	.000	.000	.425	.000	
bio17	Pearson Correlation	.214**	.186**	.214**	.818**	.346**	.983**	-.751**	.379**	1	.555**	.637**	-.215**	.026	-.197**	.041*	.228**	-.300**	-.139**	.325**	
	Sig. (2-tailed)	.000	.000	.000	.000	.000	.000	.000	.000		.000	.000	.000	.130	.000	.016	.000	.000	.000	.000	
bio18	Pearson Correlation	.376**	.351**	.344**	.636**	.505**	.521**	-.192**	.535**	.555**	1	.215**	-.107**	.227**	-.268**	.189**	.325**	-.333**	.376**	.145**	
	Sig. (2-tailed)	.000	.000	.000	.000	.000	.000	.000	.000	.000		.000	.000	.000	.000	.000	.000	.000	.000	.000	
bio19	Pearson Correlation	.222**	.060**	.316**	.847**	.719**	.602**	-.205**	.755**	.637**	.215**	1	-.152**	.287**	-.470**	-.026	.362**	-.542**	-.363**	.549**	
	Sig. (2-tailed)	.000	.000	.000	.000	.000	.000	.000	.000	.000	.000		.000	.000	.000	.121	.000	.000	.000	.000	
bio2	Pearson Correlation	.331**	.349**	.317**	-.223**	-.078**	-.226**	.281**	-.134**	-.215**	-.107**	-.152**	1	.626**	-.231**	.556**	.229**	.071**	.202**	.251**	
	Sig. (2-tailed)	.000	.000	.000	.000	.000	.000	.000	.000	.000	.000	.000		.000	.000	.000	.000	.000	.000	.000	
bio3	Pearson Correlation	.780**	.611**	.852**	.246**	.507**	-.012	.406**	.445**	.026	.227**	.287**	.626**	1	-.884**	.659**	.823**	-.712**	.287**	.693**	
	Sig. (2-tailed)	.000	.000	.000	.000	.000	.475	.000	.000	.130	.000	.000	.000		.000	.000	.000	.000	.000	.000	
bio4	Pearson Correlation	-.800**	-.576**	-.906**	-.431**	-.638**	-.158**	-.277**	-.586**	-.197**	-.268**	-.470**	-.231**	-.884**	1	-.532**	-.924**	.949**	-.198**	-.772**	
	Sig. (2-tailed)	.000	.000	.000	.000	.000	.000	.000	.000	.000	.000	.000	.000	.000		.000	.000	.000	.000	.000	
bio5	Pearson Correlation	.899**	.961**	.820**	.093**	.219**	-.010	.166**	.122**	.041*	.189**	-.026	.556**	.659**	-.532**	1	.770**	-.390**	.552**	.566**	
	Sig. (2-tailed)	.000	.000	.000	.000	.000	.575	.000	.000	.016	.000	.121	.000	.000	.000		.000	.000	.000	.000	
bio6	Pearson Correlation	.956**	.829**	.991**	.414**	.576**	.178**	.196**	.505**	.228**	.325**	.362**	.229**	.823**	-.924**	.770**	1	-.888**	.388**	.778**	
	Sig. (2-tailed)	.000	.000	.000	.000	.000	.000	.000	.000	.000	.000	.000	.000	.000	.000	.000		.000	.000	.000	
bio7	Pearson Correlation	-.731**	-.503**	-.839**	-.530**	-.673**	-.263**	-.164**	-.640**	-.300**	-.333**	-.542**	.071**	-.712**	.949**	-.390**	-.888**	1	-.161**	-.714**	
	Sig. (2-tailed)	.000	.000	.000	.000	.000	.000	.000	.000	.000	.000	.000	.000	.000	.000	.000	.000		.000	.000	
bio8	Pearson Correlation	.504**	.577**	.419**	-.097**	.030	-.177**	.178**	-.014	-.139**	.376**	-.363**	.202**	.287**	-.198**	.552**	.388**	-.161**	1	-.044**	
	Sig. (2-tailed)	.000	.000	.000	.000	.076	.000	.000	.425	.000	.000	.000	.000	.000	.000	.000	.000	.000		.009	
bio9	Pearson Correlation	.723**	.594**	.776**	.462**	.534**	.287**	.101**	.484**	.325**	.145**	.549**	.251**	.693**	-.772**	.566**	.778**	-.714**	-.044**	1	
	Sig. (2-tailed)	.000	.000	.000	.000	.000	.000	.000	.000	.000	.000	.000	.000	.000	.000	.000	.000	.000	.009		

S2-2 Correlations of the predictors in *Hyphantria cunea* in Europe (N=196)

		bio1	bio10	bio11	bio12	bio13	bio14	bio15	bio16	bio17	bio18	bio19	bio2	bio3	bio4	bio5	bio6	bio7	bio8	bio9	Note
bio1	Pearson Correlation	1	.876**	.935**	.264**	.225**	.181*	-.251**	.174*	.313**	-.233**	.264**	.337**	.541**	-.536**	.831**	.906**	-.318**	-.166*	.565**	
	Sig. (2-tailed)		.000	.000	.000	.001	.011	.000	.014	.000	.001	.000	.000	.000	.000	.000	.000	.000	.020	.000	
bio10	Pearson Correlation	.876**	1	.655**	-.021	-.067	-.011	-.362**	-.130	.095	-.399**	.016	.343**	.212**	-.067	.958**	.614**	.132	.005	.395**	
	Sig. (2-tailed)	.000		.000	.767	.348	.877	.000	.069	.185	.000	.822	.000	.003	.353	.000	.000	.066	.945	.000	
bio11	Pearson Correlation	.935**	.655**	1	.424**	.391**	.289**	-.159*	.356**	.417**	-.100	.415**	.247**	.673**	-.797**	.609**	.988**	-.608**	-.305**	.630**	
	Sig. (2-tailed)	.000	.000		.000	.000	.000	.026	.000	.000	.165	.000	.000	.000	.000	.000	.000	.000	.000	.000	
bio12	Pearson Correlation	.264**	-.021	.424**	1	.940**	.872**	-.040	.959**	.925**	.722**	.818**	.202**	.538**	-.582**	.004	.388**	-.446**	-.303**	.242**	
	Sig. (2-tailed)	.000	.767	.000		.000	.000	.581	.000	.000	.000	.000	.005	.000	.000	.961	.000	.000	.000	.001	
bio13	Pearson Correlation	.225**	-.067	.391**	.940**	1	.732**	.236**	.981**	.810**	.669**	.760**	.164*	.502**	-.574**	-.052	.354**	-.455**	-.276**	.270**	
	Sig. (2-tailed)	.001	.348	.000	.000		.000	.001	.000	.000	.000	.000	.021	.000	.000	.465	.000	.000	.000	.000	
bio14	Pearson Correlation	.181*	-.011	.289**	.872**	.732**	1	-.351**	.773**	.949**	.663**	.813**	.182*	.397**	-.397**	.018	.258**	-.283**	-.377**	.108	
	Sig. (2-tailed)	.011	.877	.000	.000	.000		.000	.000	.000	.000	.000	.011	.000	.000	.798	.000	.000	.000	.133	
bio15	Pearson Correlation	-.251**	-.362**	-.159*	-.040	.236**	-.351**	1	.217**	-.330**	.170*	-.198**	-.105	-.001	-.081	-.379**	-.156*	-.152*	.195**	-.076	
	Sig. (2-tailed)	.000	.000	.026	.581	.001	.000		.002	.000	.017	.005	.144	.993	.261	.000	.029	.034	.006	.291	
bio16	Pearson Correlation	.174*	-.130	.356**	.959**	.981**	.773**	.217**	1	.819**	.745**	.778**	.159*	.515**	-.578**	-.109	.325**	-.471**	-.258**	.232**	
	Sig. (2-tailed)	.014	.069	.000	.000	.000	.000	.002		.000	.000	.000	.026	.000	.000	.129	.000	.000	.000	.001	
bio17	Pearson Correlation	.313**	.095	.417**	.925**	.810**	.949**	-.330**	.819**	1	.588**	.858**	.208**	.465**	-.480**	.120	.385**	-.340**	-.379**	.262**	
	Sig. (2-tailed)	.000	.185	.000	.000	.000	.000	.000	.000		.000	.000	.003	.000	.000	.093	.000	.000	.000	.000	
bio18	Pearson Correlation	-.233**	-.399**	-.100	.722**	.669**	.663**	.170*	.745**	.588**	1	.372**	.133	.253**	-.198**	-.340**	-.125	-.154*	.092	-.306**	
	Sig. (2-tailed)	.001	.000	.165	.000	.000	.000	.017	.000	.000		.000	.063	.000	.005	.000	.081	.031	.201	.000	
bio19	Pearson Correlation	.264**	.016	.415**	.818**	.760**	.813**	-.198**	.778**	.858**	.372**	1	.029	.403**	-.528**	-.001	.407**	-.472**	-.579**	.520**	
	Sig. (2-tailed)	.000	.822	.000	.000	.000	.000	.005	.000	.000	.000		.691	.000	.000	.993	.000	.000	.000	.000	
bio2	Pearson Correlation	.337**	.343**	.247**	.202**	.164*	.182*	-.105	.159*	.208**	.133	.029	1	.714**	-.066	.572**	.132	.350**	.078	.036	
	Sig. (2-tailed)	.000	.000	.000	.005	.021	.011	.144	.026	.003	.063	.691		.000	.354	.000	.066	.000	.276	.616	
bio3	Pearson Correlation	.541**	.212**	.673**	.538**	.502**	.397**	-.001	.515**	.465**	.253**	.403**	.714**	1	-.727**	.353**	.611**	-.396**	-.206**	.359**	
	Sig. (2-tailed)	.000	.003	.000	.000	.000	.000	.993	.000	.000	.000	.000	.000		.000	.000	.000	.000	.004	.000	
bio4	Pearson Correlation	-.536**	-.067	-.797**	-.582**	-.574**	-.397**	-.081	-.578**	-.480**	-.198**	-.528**	-.066	-.727**	1	-.042	-.813**	.902**	.399**	-.505**	
	Sig. (2-tailed)	.000	.353	.000	.000	.000	.000	.261	.000	.000	.005	.000	.354	.000		.554	.000	.000	.000	.000	
bio5	Pearson Correlation	.831**	.958**	.609**	.004	-.052	.018	-.379**	-.109	.120	-.340**	-.001	.572**	.353**	-.042	1	.545**	.247**	.031	.345**	
	Sig. (2-tailed)	.000	.000	.000	.961	.465	.798	.000	.129	.093	.000	.993	.000	.000	.554		.000	.000	.662	.000	
bio6	Pearson Correlation	.906**	.614**	.988**	.388**	.354**	.258**	-.156*	.325**	.385**	-.125	.407**	.132	.611**	-.813**	.545**	1	-.677**	-.309**	.631**	
	Sig. (2-tailed)	.000	.000	.000	.000	.000	.000	.029	.000	.000	.081	.000	.066	.000	.000	.000		.000	.000	.000	
bio7	Pearson Correlation	-.318**	.132	-.608**	-.446**	-.455**	-.283**	-.152*	-.471**	-.340**	-.154*	-.472**	.350**	-.396**	.902**	.247**	-.677**	1	.385**	-.426**	
	Sig. (2-tailed)	.000	.066	.000	.000	.000	.000	.034	.000	.000	.031	.000	.000	.000	.000	.000	.000		.000	.000	
bio8	Pearson Correlation	-.166*	.005	-.305**	-.303**	-.276**	-.377**	.195**	-.258**	-.379**	.092	-.579**	.078	-.206**	.399**	.031	-.309**	.385**	1	-.483**	
	Sig. (2-tailed)	.020	.945	.000	.000	.000	.000	.006	.000	.000	.201	.000	.276	.004	.000	.662	.000	.000		.000	
bio9	Pearson Correlation	.565**	.395**	.630**	.242**	.270**	.108	-.076	.232**	.262**	-.306**	.520**	.036	.359**	-.505**	.345**	.631**	-.426**	-.483**	1	
	Sig. (2-tailed)	.000	.000	.000	.001	.000	.133	.291	.001	.000	.000	.000	.616	.000	.000	.000	.000	.000	.000		
